# Next-Generation Service Delivery: A Scoping Review of Patient Outcomes Associated with Alternative Models of Genetic Counseling and Genetic Testing for Hereditary Cancer

**DOI:** 10.3390/cancers10110435

**Published:** 2018-11-13

**Authors:** Jeanna M. McCuaig, Susan Randall Armel, Melanie Care, Alexandra Volenik, Raymond H. Kim, Kelly A. Metcalfe

**Affiliations:** 1 Familial Cancer Clinic, Princess Margaret Cancer Centre, University Health Network, Toronto, ON M5G 2M9, Canada; susan.randall@uhn.ca (S.R.A.); alexandra.volenik@uhn.ca (A.V.); raymond.kim@uhn.ca (R.H.K.); 2 Department of Molecular Genetics, University of Toronto, Toronto, ON M5S 1A8, Canada; melanie.care@uhn.ca; 3 Lawrence S. Bloomberg Faculty of Nursing, University of Toronto, Toronto, ON M5T 1P8, Canada; kelly.metcalfe@utoronto.ca; 4 Division of Cardiology, Department of Medicine, Peter Munk Cardiac Centre, Toronto General Hospital, University Health Network, Toronto, ONM5G 2C4, Canada; 5 Genome Diagnostics, Toronto General Hospital, University Health Network, Toronto, ON M5G 2C4, Canada; 6 Department of Medicine, Division of Medical Oncology and Hematology, University of Toronto, Toronto, ON M5S 1A8, Canada; 7 Women’s College Research Institute, Women’s College Hospital, Toronto, ON M5G 1N8, Canada

**Keywords:** BRCA, genetic counseling, genetic testing, hereditary cancer, genetic service delivery, patient outcomes

## Abstract

The combination of increased referral for genetic testing and the current shortage of genetic counselors has necessitated the development and implementation of alternative models of genetic counseling and testing for hereditary cancer assessment. The purpose of this scoping review is to provide an overview of the patient outcomes that are associated with alternative models of genetic testing and genetic counseling for hereditary cancer, including germline-only and tumor testing models. Seven databases were searched, selecting studies that were: (1) full-text articles published ≥2007 or conference abstracts published ≥2015, and (2) assessing patient outcomes of an alternative model of genetic counseling or testing. A total of 79 publications were included for review and synthesis. Data-charting was completed using a data-charting form that was developed by the study team for this review. Seven alternative models were identified, including four models that involved a genetic counselor: telephone, telegenic, group, and embedded genetic counseling models; and three models that did not: mainstreaming, direct, and tumor-first genetic testing models. Overall, these models may be an acceptable alternative to traditional models on knowledge, patient satisfaction, psychosocial measures, and the uptake of genetic testing; however, particular populations may be better served by traditional in-person genetic counseling. As precision medicine initiatives continue to advance, institutions should consider the implementation of new models of genetic service delivery, utilizing a model that will best serve the needs of their unique patient populations.

## 1. Introduction

In just over five years, multiple events have increased the demand for genetic counseling (GC) and genetic testing (GT) for hereditary breast and ovarian cancer. In 2013, Angelina Jolie’s opinion editorial in the New York Times prompted an influx of patients requesting GT of the *BRCA1* and *BRCA2* genes that was so significant that it was dubbed the “Angelina Jolie effect” [1,2]. In 2014 and 2018, the FDA approved poly (ADP-ribose) polymerase (PARP) inhibitors for *BRCA-*associated breast and ovarian cancers, respectively, which again prompted an increase in GC referrals [3], only this time with some urgency, given that results are needed to inform treatment decisions. At the same time, there has been increased awareness of the high *BRCA* mutation rates [4,5,6], and historically low genetic GC/GT rates [7,8,9,10] among women with ovarian cancer. Multi-disciplinary meetings have been convened in the United States (USA) [11] and Canada [12] to define a list of priorities to ensure that *BRCA* GT is routinely performed for all women with high-grade ovarian cancer. Additionally, the National Cancer Institute developed a “Traceback” framework to identify and provide GT to previously unreferred ovarian cancer patients and their unaffected family members [13], and the National Institute of Health recently announced a funding opportunity to support pilot research projects using this approach to identify individuals with an elevated cancer risk [14]. Ultimately, continued increases in awareness and requests for GT will have positive outcomes for hereditary cancer patients and their family members; however, there is currently a shortage of genetic counselors working in direct patient care to provide the necessary GC services [15]. The growing issue of supply and demand for GT in the field of hereditary breast and ovarian cancer requires a rapid development and implementation of alternative models of genetic service delivery.

In the traditional GC model, a health-care provider refers a patient for GC, the patient is scheduled a separate in-person appointment with a genetic counselor for pre-test GC, and results are disclosed by a genetic counselor in person or through other means [16]. While comprehensive in nature, the traditional model is time-intensive [17,18], and cancer genetic counselors using this model see fewer patients and have longer wait times compared to those who employ other models of care [18]. Alternative models of GC and GT have been developed and implemented to improve patient access; however, there remains concern regarding the quality of GC, and few genetic counselors routinely employ these models [17,18]. It is evident that models of GT where cancer patients begin with tumor testing, rather than blood testing, will soon be commonplace. Similar to the universal tumor screening model that is used in Lynch syndrome, where immunohistochemistry/microsatellite instability testing is completed automatically as part of the pathology protocol, these models would help identify patients who would benefit from GC and GT while simultaneously increasing the efficiency of genetic services. While previous review articles have described various alternative models of GC, none have considered tumor testing as an alternative model [19,20,21,22,23,24,25,26]. The purpose of this review is to provide a single comprehensive overview of patient-reported outcomes for all alternative models of GC and GT, including tumor testing, which are currently utilized for hereditary cancer to serve as a resource for clinicians to identify and implement alternative models that are relevant to their clinical resources and patient population.

## 2. Methods

A scoping review was completed to capture a wide breath of studies. Scoping reviews are considered exploratory projects that systematically map the literature that is available on a topic, and summarize a range of evidence [27,28]. They are uniquely suited to disciplines such as GC, which have emerging evidence and where there is a paucity of randomized controlled trials [27]. The methodology that was used for this review was based on the framework outlined by Arksey and O’Malley [29] and recommendations made by Levac et al. [27]. The review included five key steps: (1) identifying the research question, (2) identifying relevant studies, (3) study selection, (4) charting the data, and (5) collating, summarizing, and reporting the results. The review did not complete the optional sixth step of completing a consultation exercise.

### 2.1. Identifying the Research Question

The review was anchored in the following question: “What are the alternative models of pre-test GC and GT that are used for hereditary cancer, and what is the patient impact of employing these alternative models of care?” For the purposes of this review, any model that did not involve a separately scheduled appointment for an in-person pre-test GC from a genetic counselor or medical geneticist was considered an alternative model of care.

### 2.2. Identifying Relevant Studies

To conduct a wide review, relevant studies were identified from seven databases: Medline, Epub Ahead of Print, Embase, PyschINFO, CINAHL, the Cochrane Database of Systematic Reviews, and the Cochrane Central Register of Controlled Trials. A librarian at the University Health Network was consulted to develop a search strategy (Appendix A). MeSH headings and keywords were used to combine cancer terms (including “Neoplasms”, “cancer”, “tumor”, “HBOC” (hereditary breast/ovarian cancer), “HNPCC”, “Lynch”) with genetic terms (including “Genetic Services”, “Genetic Counseling”, “Genetic Testing”, “germline test”, “molecular screen”, “universal screen”, “somatic test”) and patient outcomes terms (including “Patient Outcome Assessment”, “Patient Reported Outcome Measures”, “Patient Satisfaction”, Stress Psychological”, “access”). Results were limited to English articles published ≥2007 and conference abstracts published in ≥2015. An initial search conducted on 22 November 2017, and an updated search was completed on 6 June 2018. The search strategy identified a total of 14,993 articles for review. Citations were imported into EndNote citation software (Clarivate Analytics; Boston, MA, USA) for manual de-duplication. The resulting 10,270 articles were imported into a web-based citation screening platform, Covidence (Veritas Health Innovation Ltd.; Melbourne, AUS), where an additional 538 duplicates were removed, leaving a total of 9732 articles for review.

### 2.3. Study Selection

**Figure 1:**
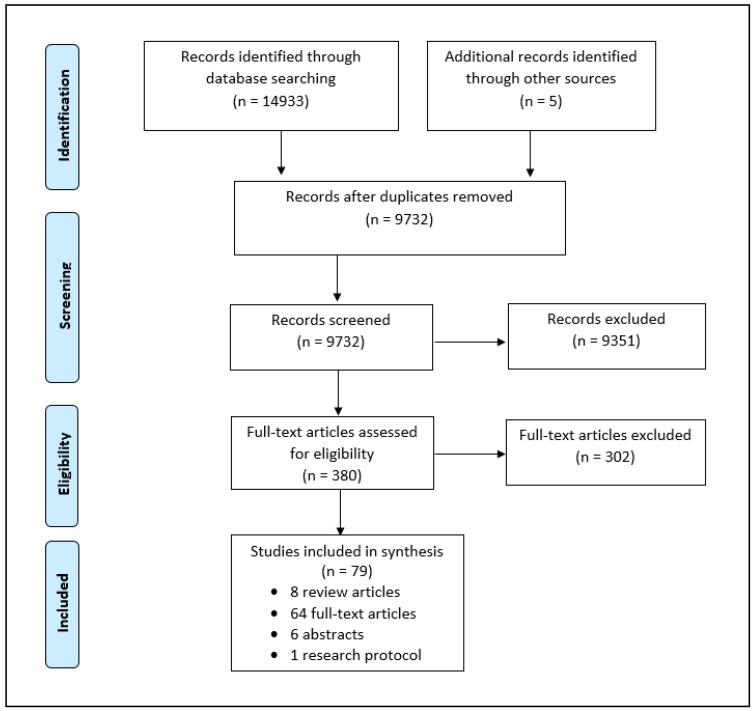
Prisma Flow Diagram.

Inclusion and exclusion criteria were developed by the study team (Appendix B). Articles were included if they assessed the patient outcomes of adults receiving GT or GC for hereditary cancer using alternative models of care. Published protocols for ongoing studies and conference abstracts without full-text publications were also included. Due to limited resources, articles were excluded if they were not available in English. To ensure the relevancy of information, articles published <2007 and conference abstracts published <2015 were excluded. Additional exclusion criteria included studies involving individuals at a 50% risk of a hereditary cancer syndrome, direct to consumer GT, tumor GT for somatic targets only, GT for non-cancer indications, and articles that did not provide a direct assessment of patients’ experiences or outcomes. All of the articles were screened by the first author (J.M.M.) and one of three secondary reviewers (S.R.A., A.V., M.C.). All of the reviewers have at least five years of GC experience. Conflicts were resolved through discussion amongst reviewers. When a resolution could not be achieved, an independent third reviewer (K.A.M.) was consulted. The search strategy results identified 74 relevant publications. Screening the reference lists from eight selected review articles [19,20,21,22,23,30,31,32] identified an additional five articles, resulting in a total of 79 publications (Figure 1).

### 2.4. Charting the Data and Reporting Results

Data charting and extraction was completed by J.M.M. and recorded using a data extraction form. Extracted data included article title, authors, country, publication year, study population, details of GC/GT intervention, study design, and patient outcomes measured. Articles were grouped by invention type, and the data extraction form was used to summarize and report the results.

## 3. Results

A total of 79 publications (8 review articles, 6 conference abstracts, 1 protocol, and 64 full-text articles) describing 69 studies were included in this review. Five articles were qualitative in nature. Studies were grouped into seven categories based on the GC intervention (Table 1, Table 2, Table 3, Table 4, Table 5, Table 6 and Table 7), which can be dichotomized into two groups: those that involved a genetic counselor prior to GT (alternative GC models: telephone, telegenetic, group, and embedded GC models), and those that did not (alternative GT models: mainstreaming, direct GT, and tumor-first GT models). Among publications of alternative GC models, the most common outcomes that were examined were patient satisfaction (*n* = 16), psychosocial outcomes (*n* = 13), knowledge (*n* = 13), and uptake of GT (*n* = 13). Of those reporting on alternative GT models, the most common outcomes discussed were the uptake of GT (*n* = 25), psychosocial outcomes (*n* = 19), attendance at GC (*n* = 17), and referral for GC (*n* = 16). While not a specific outcome of interest for this review, a cost reduction was noted in the telephone (*n* = 1) telegenetic (*n* = 1), group (*n* = 2), mainstreaming (*n* = 1), and direct GT (*n* = 2) models. Detailed information about each model is provided in the following sub-sections.

### 3.1. Telephone Genetic Counseling Models

Telephone GC models involve the provision of GC completely by telephone, and may involve mailed resources provided to patients in advance of their appointments. Eight articles were identified, representing four separate studies conducted in the USA (Table 1) [33,34,35,36,37,38,39,40]. All eight studies involved GC for hereditary breast/ovarian cancer. Two articles described patient experience with telephone GC without comparison to traditional GC, and the remaining six articles reported results from two randomized non-inferiority trials comparing telephone and traditional GC models. All of the studies demonstrated improved patient knowledge following telephone GC. Both non-inferiority trials demonstrated telephone GC to be non-inferior to traditional GC on knowledge, psychosocial, and counseling measures [35,36,39]. The Schwartz trial also noted a cost benefit of telephone GC by comparing estimates of patient, clinician, testing, and overhead costs [36]. In assessments of patient satisfaction, those receiving telephone GC were more likely to report that GC was convenient; however, they were less likely to report having no difficulty in maintaining attention or perceiving a high level of emotional support/recognition [38]. Most women were very satisfied with telephone GC; however, these results may be biased, as one-third of patients declined participation in the trial, citing a preference for in-person GC [36]. Unfortunately, both non-inferiority trials reported lower, non-equivalent rates of GT in the telephone group. Follow-up studies suggest that ethnicity, cancer-specific distress, and perceived mutation risk may moderate patient decisions to pursue GT, where ethnic minorities and patients with higher levels of distress or perceived risk were less likely to pursue GT after telephone, rather than traditional GC [37,40]. 

### 3.2. Telegenetic Genetic Counseling Models

In telegenetic GC models, GC is provided remotely via videoconference. Eight articles were identified, representing one Australian study with separate publications for quantitative and qualitative analyses, and six North American studies (Table 2) [41,42,43,44,45,46,47,48]. All eight studies involved GC for hereditary cancer, two of which focused on breast/ovarian cancer. In contrast to other studies where genetic counselors and/or geneticists are located remotely, the Australian telegenetic GC model involved a telegenetic appointment with a remote geneticist and a local genetic counselor [42,43]. Qualitative analyses suggest that patients are satisfied with telegenetic GC, but this model may be best suited for patients seeking information rather than emotional support [42]. Quantitative analyses comparing traditional and telegenetic GC models observed no significant differences in knowledge, satisfaction, or measures of anxiety and depression; however, telegenetic GC was significantly better at meeting patients’ expectations and promoting perceived personal control [43]. Only 7% of patients receiving telegenetic GC would have preferred a face-to-face appointment [43]. Among North American studies, three assessed telegenetic GC alone [41,44,46] and three compared telegenetic GC to traditional GC, including one randomized trial [45,47,48]. Knowledge significantly increased after telegenic GC [46,48]. In three studies assessing psychosocial outcomes, levels of anxiety, depression, and worry about developing cancer, all decreased after telegenetic GC [46,47,48]. All six North American studies reported high levels of patient satisfaction with telegenetic GC; however, a third of patients would have preferred in-person GC [44,45,48], and almost a quarter of patients in one study declined a telegenetic appointment, preferring to schedule an in-person appointment [41]. Buchanan et al. also reported lower attendance rates for telegenic compared to in-person GC appointments [45]. One study demonstrated telegenetic counseling to be a cost-effective alternative to outreach GC by comparing the total cost of telegenic GC (GC service + telegenetic set-up and support) to the total cost of outreach GC (GC service + travel time + mileage) [45]. In other centers, the travel costs may be borne by patients who are required to travel to the genetics clinic.

### 3.3. Group Genetic Counseling Models

Group GC models involve an informative group session that may or may not be followed by brief one-on-one GC. Six articles were identified, including four North American studies and two European studies (Table 3) [49,50,51,52,53,54]. All six used group GC models for hereditary breast and ovarian cancer, two of which were limited to Ashkenazi Jewish individuals [52,54]. Four articles compared group GC to traditional GC [50,51,52,53]. Importantly, a non-inferiority trial assessing the use of a group DVD GC session followed by individualized GC demonstrated group GC to be non-inferior with respect to knowledge, risk perception, and GC satisfaction measures [52]. Three additional studies assessed knowledge scores [49,51,53], and only Rothwell et al. did not observe significantly improved knowledge scores; however, this was attributed to high baseline knowledge scores [51]. Rothwell et al. also assessed psychosocial outcomes, and found that both traditional and group GC increased perceived personal control, decreased cancer-specific distress, and improved depression, with no significant differences between the two groups [51]. All six studies reported high levels of patient satisfaction with group GC. Two studies found that many women prefer individual to group GC [50,51]; however, in a retrospective study of low-risk Ashkenazi Jewish individuals, only 8% reported a preference for traditional GC [54]. Time was assessed in five studies [49,50,51,52,53], and cost was assessed in two [49,52]. In studies providing direct comparisons to a traditional GC model, notable time-saving was reported for the group GC model [51,52,53]. Two studies extrapolated the GC time to demonstrate a cost benefit to the group GC [49,52]. Two studies examining the uptake of GT reported discrepant results, with Rothwell et al. reporting a significantly lower uptake after group GC [51], and Manchanda et al. reporting equivalent GT rates [52].

### 3.4. Embedded Genetic Counseling Models

In the embedded GC model, genetic counselors are integrated into oncology clinics, where genetic counselors provide genetic counseling and facilitate genetic testing during oncology visits. Additional roles may also include genetic risk assessment, the identification of appropriate genetic referral, referral triage, and clinician education. One Australian and three American studies were identified (Table 4) [55,56,57,58]. All four studies reported improvements in GC referral rates for breast or ovarian cancer patients. While Senter et al. reported an overall increase in GC referral and patient follow-through, the highest rates were recorded when genetic counselors and oncologists were in the same oncology clinic on the same day [56]. In addition to improved referral rates, all of the studies noted improved efficiencies, such as reduced patient wait times for GC, and shorter GC sessions. Importantly, Pederson reported that the reduced wait times increased the likelihood for patients to have GT prior to breast surgery, resulting in a decrease in the time to treat breast cancer patients [58].

### 3.5. “Mainstreaming” Genetic Testing Models

Popularized by the *Mainstreaming Cancer Genetics Program* in the United Kingdom (UK) [59], mainstreaming GT models engage non-genetics clinicians to order GT, typically with support from genetic clinicians. Referrals to clinical genetic services are processed in the event of positive or inconclusive GT results. This model has also been referred to as a *Triage* GC model [16]. Five publications were identified: two from the UK, one from the USA, one from an international study, and one abstract from a Malaysian study (Table 5) [57,59,60,61,62]. All five studies assessed GT of the *BRCA1* and *BRCA2* genes. Patient satisfaction with the mainstreaming model was high, and patients were glad to have had GT organized during their oncology visits [59,61]. The uptake of GT with this model was high, potentially improving access to GT. In their study, Bednar et al. reported that over half of ovarian cancer patients at a regional clinic in the USA received GT via their physician, many of whom may not have otherwise had access to GT [57]. Following positive results, the rates of GC referral [59,62] and patient attendance for GC [62] were high; however, Rahman et al. reported that only 22% for women with a variant of uncertain significance were referred for GC [62]. Considering the reduced number of GC appointments required in the mainstreamed GC model, one study estimated that this model provides a fourfold reduction in patient wait times and 13-fold reduction in health-care costs [59]. Yoon et al. was the only study using a validated tool to assess the psychosocial impact of mainstreamed GT. While distress levels decreased after post-test GC, 17% of patients still required additional support. Distress may be attributed to their cancer diagnosis, rather than GT results, as most had distress related to living with cancer, and about half frequently worried about getting cancer again [60].

### 3.6. Direct Genetic Testing Models

In direct GT models, patients are offered GT with limited to no pre-test discussion. Written documents or other resources may be provided in lieu of GC. Fifteen articles, representing nine studies from six countries, were identified (Table 6) [63,64,65,66,67,68,69,70,71,72,73,74,75,76,77]. Although not in the context of a research study, Brierly et al. explored a series of cases where pre-test GC was not provided by genetics professionals, and cautioned of the negative outcomes of this model, including unnecessary prophylactic surgeries, unnecessary testing, psychosocial distress, and false reassurance resulting in inappropriate medical management [63]. These concerns were not evident in the results of the 14 remaining articles. All 14 articles involved GT of the *BRCA1* and *BRCA2* genes.

Two studies (four articles) focused on providing direct GT to individuals of Ashkenazi Jewish descent, including one qualitative study [64,65,76,77]. Satisfaction with the direct GT model was high among *BRCA* mutation carriers and non-carriers, and the majority would recommend this model of GT to others [64,76]. All of the women who were identified to have a *BRCA* mutation attended an in-person post-test GC [64,76], and compliance with high-risk screening and risk-reducing surgeries was high [65]. With respect to psychosocial measures, levels of cancer-specific distress following GT were significantly higher among *BRCA* mutation carriers [64,76]; however, in a two-year follow-up study of *BRCA* mutation carriers, Metcalfe et al. found that levels of distress decreased significantly, especially among women who pursued risk-reducing surgeries [65]. With respect to knowledge, cancer genetics knowledge levels increased only among those who received in-person post-test GC (*BRCA* carriers and non-carriers with a family history) [76]. Two additional studies, which were not limited to the Ashkenazi Jewish population, included women with and without a personal diagnosis of cancer [66,67]. *BRCA* mutation carriers reported longer GC sessions and a higher uptake of risk-reducing mastectomy or breast MRI when counseling was provided by a genetic professional [66]. Reported levels of knowledge and satisfaction were higher among those who reported having pre-test GC with a genetic counselor [67].

The remaining four studies (eight publications) included women diagnosed with ovarian cancer [70,71], breast cancer [68,69,72,73], or both [74,75]; qualitative and quantitative analyses were published for each patient group. The qualitative data suggested that the impact of GT was minimal in comparison to a cancer diagnosis [71,72,75]; however, some women still reported a preference for discussing GT with a health-care provider prior to GT [75]. The decision to pursue GT was often motivated by altruistic factors [71,75] and to inform treatment [72]. Among five quantitative publications, two involved patients receiving direct GT [70,74], and three compared direct GT to the traditional GC model, including a randomized non-inferiority trial [68,69,72,73]. Congruent with the qualitative data, the quantitative data demonstrated that levels of distress, depression, and anxiety were lower in response to GT than in response to an ovarian cancer diagnosis [70]. The average level of depression and anxiety among newly diagnosed breast/ovarian cancer patients pursuing GT was comparable to that of breast and gynecologic cancer patients in general [74]. The results of the non-inferiority trial demonstrated that the direct GT model was non-inferior to the traditional GC model on measures of decisional conflict about GT, knowledge, and psychosocial measures [73]. Patient satisfaction and acceptability of the direct GT model was high. In a patient preference study, no differences in the quality of life, breast cancer worry, or risk perceptions were noted in women who chose direct GT or traditional GC; however, levels of decisional conflict were higher, and the uptake of GT was lower, among women who chose traditional GC [68,69]. Considering the costs associated with GC services, two studies reported a cost benefit of direct GT models, as compared to traditional GC [70,73].

### 3.7. Tumor-First Genetic Testing Models

In tumor-first GC models, genetic screening is first performed on a sample of tumor tissue, often as part of a pathology workflow, with GC offered based on the tumor results. A total of 26 publications from six countries that reviewed patient outcomes of tumor testing were identified (Table 7) [78,79,80,81,82,83,84,85,86,87,88,89,90,91,92,93,94,95,96,97,98,99,100,101,102,103]. In total, 23 publications [78,79,80,81,82,83,84,85,86,87,88,89,90,91,92,93,94,95,96,97,98,99,100], including four abstracts [90,92,93,96], assessed patient outcomes of immunohistochemistry and/or the microsatellite instability testing of tumor tissue as an initial screening test for Lynch syndrome. Ten out of 15 studies reporting the rate of GC referral following abnormal tumor screening for Lynch syndrome reported a 100% GC referral rate [79,80,85,86,88,90,94,96,99,100]; however, one study reported an 18% referral rate [82]. Referral rates increased when a genetic counselor or navigator was directly involved in the screening protocol, and when patients had additional features suggestive of Lynch syndrome [79,82,86,100]. In a study assessing the role of ethnicity, Kupfer et al. reported high rates of GC referral and GT in Caucasian patients [92]. A total of 14 studies reported the uptake of GC among patients who screened positive for Lynch syndrome [79,80,81,82,83,85,86,88,89,90,95,96,99,100]. The majority of patients who were referred for GC attended an appointment. Three studies reported low GC rates (30–42%); however, it is unclear whether patients had been contacted [83] or referred [89,100]. Of patients who attended GC, >70% pursued GT [79,80,81,82,83,85,88,90,93,96,97,98,100]. Four studies reported on the psychosocial impacts of tumor GT [78,84,87,91]. In a model where tumor results were disclosed directly to patients via a patient portal, satisfaction was high, anxiety levels did not change immediately after tumor results, and 80% of patients with abnormal tumor results pursued GC/GT [84]. Patient attitudes about tumor testing were also generally positive [87,91]. When surveyed prior to tumor results, patients reported low levels of distress [78,87]; however, one study found that 40% of patients with abnormal tumor results had high levels of cancer-related distress, which subsequently decreased over time [78].

The remaining three publications assessed the use of DNA sequencing of tumor tissue for patients with advanced disease to identify potential druggable targets. Two studies examining patient preferences reported positive patient attitudes toward tumor testing; however, neither study directly reported data on the patient impact of receiving tumor genetic test results [101,102]. The published protocol of a mixed-methods longitudinal study outlined ongoing research to evaluate patient knowledge and preferences, as well as the behavioral, decisional, and psychological outcomes of tumor testing in individuals with advanced cancer diagnoses [103].

## 4. Discussion

The purpose of this scoping review was to first determine the various alternative models of pre-test GC and GT used for hereditary cancer, and second to determine the patient impact of employing these models of care. Seven alternative models of care were identified and dichotomized into alternative GC models (telephone, telegenetic, group, embedded GC models) and alternative GT models (mainstreaming, direct, and tumor-first GT models). The results of this review provide insight into rates of GC/GT, patient preferences, and psychosocial outcomes associated with alternative models of genetic service delivery, which can be carefully evaluated by the clinicians who are considering utilizing these models. While not an outcome of interest for this review, the cost–benefit reported in several studies may be an important consideration for policy makers. 

### 4.1. Alternative GC Models

The results of this scoping review indicate that all four alternative models of GC can improve patient access to genetic services; however, this may not necessarily translate into an increased uptake of GC and GT. Both telephone and telegenetic models extend the reach of genetic counselors by eliminating or reducing the travel required to access genetic services. Despite this advantage, a lower uptake of GC and GT has been identified in telegenetic and telephone models, respectively. Data suggests that low rates of GT among telephone GC may be related to ethnicity [37] and high cancer-specific distress or perceived hereditary cancer risk [40]. Group GC can also improve patient access to genetic services by increasing the efficiency of genetics clinics and allowing genetic counselors to see higher volumes of patients; however, patient uptake of GC with the model may be low [50], and there is conflicting data regarding patient uptake of GT following group GC [51,52]. Finally, the embedded GC model may improve patient access to GC and GT by improving GC referral rates and facilitating GC/GT during oncology appointments. Based on the small number of studies included in this review, the embedded GC model appears to improve the patient uptake of both GC and GT.

While it is important to improve patient access to genetic services, it is paramount that patient experience is considered when implementing alternative GC models. The studies in this review, including four non-inferiority studies, suggest that telephone, telegenetic, and group GC models improve patient knowledge and psychosocial functioning. While patients appear to be satisfied with these alternative models, some patients may still prefer the traditional GC model. It is important to consider these results in the context of the potential bias created by patients who declined study participation in order to receive traditional GC, as reported in studies of telephone [36], telegenetic [41], and group [50] GC models. Studies evaluating the embedded GC model did not examine its psychosocial impact on cancer patients, demonstrating the need for future research regarding patient psychosocial outcomes associated with this model of care.

Overall, the four alternative GC models may be useful in increasing patient access to genetic services; however, they may not be appropriate for all patients. The combination of patient preference data and reports of lower GT rates among specific patient groups indicate that traditional GC should remain available as an option to patients, particularly for those seeking emotional support during the GT process.

### 4.2. Alternative GT Models

By eliminating pre-test GC with a genetic counselor and facilitating GT outside of the genetics clinic, alternative GT models undoubtably improve patient access to genetic information. Indeed, the small number of included studies evaluating mainstreaming GT models reported a high patient uptake of GT. It is concerning that one study that reported patients with positive or inconclusive results may not be referred for GC in a timely manner, or at all [62]. While many studies of direct GT models evaluated the psychosocial impact of direct GT models, few reported on the patient uptake of GT and post-test GC. The data from one study suggested that patient uptake may be higher with a direct GT model as compared to the traditional GT model; however, participants were not randomized to a specific GT model, and this discrepancy may be attributed to patient characteristics rather than the model itself [68]. Additionally, patients receiving negative GT results may be less likely to attend post-test GC recommended based on family history [76]. Most studies assessing GC referral, GC attendance, and the uptake of germline GT after tumor testing to screen for Lynch syndrome reported high uptake of these services; however, low rates have also been reported [82,89,92,95,99]. The data from this review demonstrates that facilitation by a genetic counselor or navigator results in a higher uptake of GC and germline GT, suggesting that some central oversight may be required when implementing tumor-first GT models.

The results of this review indicate that patient satisfaction with alternative GT models is high; however, additional data may be required to further evaluate patient psychosocial outcomes. Only one study involving mainstreaming GT employed validated tools to assess the psychosocial impact of this model, suggesting that further research is needed. In contrast, multiple quantitative and qualitative studies of the direct GT model evaluated its psychosocial impact. While this model does not appear to cause increased distress or anxiety among cancer patients, some studies suggest that a subset of women may benefit from having GC prior to GT, including those with higher levels of decisional conflict [68], baseline distress levels [68], unaffected individuals [64], and those requiring additional emotional support throughout the GT process [75]. While only four studies evaluating tumor screening for Lynch syndrome reported on psychosocial outcomes, this model does not appear to have a negative impact on patients. Based on this review, it seems that cancer patients are interested in tumor GT; however, included studies did not assess the patient impact of tumor GT outside of screening for Lynch syndrome. As with the mainstreaming GT model, additional research regarding the psychosocial outcomes of tumor-first GT models is required.

The three alternative GT models included in this review have the potential to improve patient access to GT. Yet, some patients may prefer to receive GC prior to GT. As such, further research is required to evaluate the psychosocial outcomes associated with mainstreaming and tumor-first GT models. In addition to those with positive GT results, patients with negative or inconclusive results may also benefit from post-test GC, as they can experience increased distress or misinterpret the clinical implications of their results [104,105]. As cautioned by Brierley et al., care must also be taken to ensure that patients receiving negative test results from mainstreaming, direct GT, or tumor-first models are not falsely reassured [63]. Thus, patients with negative GT results and a relevant history of cancer should be referred for further genetic evaluation. The implementation of alternative GT models will likely require “buy-in” from all of the stakeholders, and should involve educational training for ordering clinicians.

### 4.3. Future Directions

As targeted therapies continue to develop, more emphasis may be placed on tumor-first models to streamline GT processes. By reducing the reliance on clinicians to refer patients for GC, or order GT themselves, tumor-first models may ensure equal and rapid access to genetic information for all patients. Since DNA sequencing of tumor tissue can identify both germline and acquired pathogenic variants, this model can both inform treatment and identify the subset of patients who may have a hereditary cancer risk and warrant a referral for GC and targeted GT. With the rare exception of gene reversions, where pathogenic variants revert to wild-type in response to treatment, negative genetic tumor tests correlate with negative germline results. Therefore, only those patients with positive tumor results, negative tumor results with a suggestive family history, or negative tumor results in pre-treated tumor tissue, need to be seen for GC. Based on studies of alternative GT models, individuals with abnormal results have high rates of follow-through for post-test GC, which allows genetic counselors to provide information and support as well as discuss the familial implications of their result, creating opportunities for cancer prevention in at-risk relatives. As evidenced by this review, there is currently no data on the patient impact of automatic DNA sequencing of tumor tissue at the time of cancer diagnosis. While data from a limited number of studies involving tumor screening for Lynch syndrome suggests that automated tumor testing does not have a negative effect on patients, the immunohistochemistry and microsatellite instability testing conducted for Lynch syndrome may be perceived differently by patients and clinicians than DNA sequencing of tumor tissue. Thus, the reported patient outcomes associated with tumor testing in Lynch syndrome may not be indicative of patient outcomes associated with other tumor GT models. Likewise, the available data on the DNA sequencing of tumor tissue is comprised of patients with advanced stage disease, whose motivations and perceptions of GT may be vastly different from individuals with early-stage disease. As tumor GT models are implemented earlier in a patient’s treatment cycle, it will be critical to assess the impact of these models with respect to the timing and stage of the disease.

### 4.4. Study Limitations

The results of this review should be considered in the context of several limitations. First, it is not a systematic review, which is often considered the gold standard for reviewing available literature. Many of the included articles were qualitative in nature (*n* = 5), non-randomized studies comparing alternative and traditional strategies (*n* = 13), or did not provide a comparison to traditional strategies (*n* = 44). Despite their potential lack of rigor, the articles provide valuable insights for genetic counselors considering changes to their practice, and would have been missed in a systematic review. Second, as is the norm for scoping reviews, a quality assessment was not completed for the included articles. Third, scoping reviews have been criticized for their oversimplification and potential to mask variation between studies [106]. By grouping studies by model of service delivery, this review does not consider the nuances in study design and patient populations. The educational and emotional needs of individuals with and without cancer, as well as those diagnosed with breast, ovarian, or colon cancer, are vastly different, and were not assessed separately during this review. Fourth, the majority of the published literature regarding the tumor-first GT model has been conducted in the context of Lynch syndrome, and the patient perspective of immunohistochemistry and microsatellite instability testing may not translate to patient perspectives of the DNA sequencing of tumor tissue. Likewise, the few perspectives obtained from patients with advanced disease may not be applicable to those with a new cancer diagnosis. As tumor GT becomes part of routine care for cancer patients, further research is necessary to determine its impact on patients and families. 

## 5. Conclusions

The field of hereditary cancer has changed significantly since the discovery of the *BRCA1* and *BRCA2* genes in the mid-1990s, and it is important that models of genetic service delivery evolve as well. Alternative models of GT and GC are unlikely to ever be as comprehensive as the traditional GC model; however, in the current era of precision medicine, many patients may be better served by models that increase their access to GT or GC, either by minimizing the number of in-person appointments or reducing clinical wait times. Yet, it remains important for providers to recognize that alternative models of GT and GC are not appropriate for, or acceptable to, all patients or clinical situations. The traditional GC model is likely to continue to have a role in GT for hereditary cancer, particularly for individuals without a personal diagnosis of cancer, or those requiring greater emotional support. It is evident from this review that there is no “one size fits all” approach that will suit every patient, clinician, or institution. The benefits and limitations of each model should be evaluated from the lens of each stakeholder when deciding which models of care to utilize. As GT continues to evolve, moving from the germline to tumor GT, new and exciting opportunities will emerge, allowing genetic counselors to expand their current roles, integrate GC into the field of oncology, and explore new models of genetic service delivery.

## Figures and Tables

**Table 1:** Telephone genetic counseling models.

Study	Country	Population	Selected Outcomes: GC Referral Rates [A], GC Attendance [B], GT Uptake [C], Wait Time [D], GC Time [E], Satisfaction [F], Knowledge [G], Psychosocial [H], Health Behaviors [I], Patient Preferences [J]
Pal et al. (2010) [33]	USA	African American women with BC ≤ 50 receiving telephone GC with tailored counseling aid (*n* = 37)	There was a significant increase in knowledge after GC. [G] Compared to a counseling aid and a personalized letter, telephone GC was the most helpful tool in enhancing knowledge. [G]
Sutphen et al. (2010) [34]	USA	Health insurance company employees receiving telephone GC after screening positive for HBOC risk (*n* = 39)	All employees who screened positive had telephone GC. [B] All individuals/families who were eligible had GT. [C] Patients reported the genetic counselor understood their stress (81%), spent the right amount of time with them (81%), and communicated an effective cancer risk-management plan (>70%). [F] >90% reported improved knowledge after GC. [G] 87% planned to engage in behaviors to reduce their cancer risks. [I]
Kinney et al. (2014) [35] *	USA	Randomized trial of rural and urban women aged 25–74 years with a personal/family history suggestive of HBOC who had telephone (*n* = 437) or traditional (*n* = 464) GC	Uptake of GT was not equivalent, with fewer patients having GT after telephone (22%) vs. traditional (32%) GC. [C] Telephone was non-inferior to traditional GC on measured outcomes one week after pre-test GC (knowledge, anxiety, cancer-specific distress, patient-centeredness) and six months after the last GC (anxiety, cancer specific distress, decisional conflict, decisional regret, quality of life). [G, H]
Schwartz et al. (2014) [36] **	USA	Randomized trial of women aged 21–85 years with ≥10% risk of a *BRCA* mutation who had telephone (*n* = 298) or traditional (*n* = 302) GC	Uptake of GT was not equivalent, with fewer patients having GT after telephone (84%) vs. traditional (90%) GC. [C] Telephone was non-inferior to traditional GC on measured outcomes two weeks after pre-test GC (knowledge, decisional conflict, cancer-specific distress, perceived stress, GC satisfaction), and three months after post-test GC (decisional conflict cancer specific distress, perceived stress, physical and mental function). [G, H] 122 patients declined the study because they wanted traditional GC. [J] Cost per patient for telephone GC was $114.40 less than traditional GC.
Butrick et al. (2015) [37] **	USA	Randomized trial of women aged 21–85 years with ≥10% risk of a *BRCA* mutation who had telephone (*n* = 298) or traditional (*n* = 302) GC	Most women who had GC (84% telephone; 90% traditional) had GT. [C] High GT uptake among women who were married (OR = 1.85), had higher objective risk (OR = 1.22), higher knowledge (OR = 1.13), or received traditional GC (OR = 1.65). [C] Ethnicity was the only moderator of GT uptake on GC model, with fewer minority women having GC in the telephone group (OR = 0.41). [C]
Peshkin et al. (2015) [38] **	USA	Randomized trial of women aged 21–85 years with ≥10% risk of a *BRCA* mutation who had telephone (*n* = 272) or traditional (*n* = 282) GC	Most were satisfied with telephone (83%) and traditional (87%) GC. [F] Telephone GC group was more likely rate GC as highly convenient (OR = 4.78). [F] Telephone GC group were less likely to report no difficulty maintaining attention (OR = 0.46), or that the counselor was effective in providing support (OR = 0.56) and recognizing emotions (OR = 0.53). [F] Cancer distress moderated difficulty maintaining attention in the traditional group (OR = 0.71); race/ethnicity moderated perceived counselor support, with a significant increase in support for Caucasian women in the traditional group (OR = 3.11). [F] Most women preferred the method of GC that they received, or had no preference (81% telephone; 84% traditional). [J]
Kinney et al. (2016) [39] *	USA	Randomized trial of rural and urban women aged 25–74 years with a personal or family history suggestive of HBOC who had telephone (*n* = 409) or traditional (*n* = 383) GC	Uptake of GT was not equivalent, with fewer patients having GT after telephone (28%) vs. traditional (37%) GC. [C] Highest rate of GT was in rural patients having traditional GC (41%). [C] One year after GC, telephone was non-inferior to traditional methods on measured outcomes (anxiety, cancer-specific distress, decisional conflict, perceived personal control, quality of life, decisional regret). [H] 3/20 BRCA+ women with ≥1 breast prior to GT had risk reducing mastectomy (1/10 telephone; 2/20 traditional). [I] 6/10 *BRCA*+ women ≥35 years old with ≥1 ovary had risk-reducing oophorectomy (2/4 telephone; 4/6 traditional). [I]
Steffen et al. (2017) [40] *	USA	Randomized trial of rural and urban women aged 25–74 years with known mutation status who had telephone (*n* = 402) or traditional (*n* = 379) GC for HBOC	Independent predictors of GT were higher cancer-specific distress (OR = 1.01), higher perceived comparative mutation risk (OR = 1.32), lower ratings of genetic counselor patient-centeredness (OR = 0.45), and an absence of short-term (OR = 18.73) and long-term (OR = 6.64) cost barriers. [C] Moderators of GT uptake in the telephone group were high psychological distress (OR = 0.45 vs. 0.86) and perceived comparative mutation risk (OR = 0.50 vs. 0.74). [C, H]

GC = genetic counseling; GT = genetic testing; OC = ovarian cancer; BC = breast cancer; HBOC = hereditary breast/ovarian cancer * publications from the same study; ** publications from the same study.

**Table 2:** Telegenetic genetic counseling models.

Study	Country	Population	Selected Outcomes: GC Referral Rates [A], GC Attendance [B], GT Uptake [C], Wait Time [D], GC Time [E], Satisfaction [F], Knowledge [G], Psychosocial [H], Health Behaviors [I], Patient Preferences [J]
d’Agincourt-Canning et al. (2008) [41]	CAN	Patients (*n* = 43) and family members (*n* = 5) having telegenetic GC for hereditary cancer	Satisfaction was high, with patients feeling they could communicate, ask questions, and understand the information; reported advantages were cost-savings, convenience, presence of family. [F] 10 patients declined telegenetic GC, preferring traditional GC. [J]
Zilliacus et al. (2010) [42] *	AUS	*Qualitative study* of women who received GC for HBOC, where a genetic physician provided telegenic GC with a local genetic counselor present in-person (*n* = 12)	75% would have telegenetic GC again; 58% wanted to meet the physician in person. [F] 83% reported no technical difficulties, 92% wanted GC for information rather than support, and 92% felt that the clinician was approachable. [F] Reported advantages included a reduction in travel and associated costs; one woman found the appointment impersonal. [F]
Zilliacus et al. (2011) [43] *	AUS	Patients receiving traditional (*n* = 89) or telegenetic (*n* = 106) GC for HBOC, where a genetic physician provided telegenic GC with a local genetic counselor present in-person	Telegenetic GC was equivalent to traditional GC regarding cancer-specific anxiety. [H] No difference between the groups with respect to changes in knowledge scores, generalized anxiety, or depression. [G.H] Telegenetic group had a higher increase in perceived personal control; there was no difference in perceived clinician empathy. [H] Satisfaction was high in both groups; telehealth group had a higher level of expectations being met. [F] Of those receiving telegenetic GC, 7% preferred face-to-face GC, 33% preferred telegenetic, and 59% had no preference. [J]
Meropol et al. (2011) [44]	USA	Patients and families receiving telegenetic GC for hereditary CRC or HBOC (*n* = 31)	Patients had high levels of satisfaction with technical, information, communication, and psychosocial components; all patients would recommend telegenetic GC to others. [F] 22% of patients felt uncomfortable, and 10% felt telegenetic GC was impersonal. [F] 29% would have preferred face-to-face GC. [J]
Buchanan et al. (2015) [45]	USA	Randomized trial of individuals receiving telegenetic (*n* = 59) or traditional (*n* = 71) GC for hereditary cancer risk.	Patients in the traditional GC group were more likely to attend GC (89% vs. 79%). [B] No significant difference in GT uptake between telegenetic (54%) and traditional (55%) GC. [C] Visit and GC specific satisfaction was high for both telegenetic and traditional GC. [F] 98% who had telegenetic GC were comfortable with the model; 32% would have preferred traditional GC. [J] The cost of telegenetic GC ($106.19) was lower than traditional ($244.33) GC.
Bradbury et al. (2016) [46]	USA	Patients >20 years eligible for GT (HBOC or CRC) receiving telegenic GC (*n* = 61)	30.4% had disconnections and 3.9% of appointments failed due to technology issues. Average pre-test GC was 61 min, and post-test was 25 min. [E] Advantages included reduced travel burden and convenience; no disadvantages were reported. [F] Overall satisfaction was high; among those who had GT, satisfaction with telegenetic and genetic services significantly increased after post-test GC. [F] Knowledge significantly increased after GC. [G] There was a significant decrease in anxiety and depression after pre-test and post-test GC, but no change in state anxiety or cancer worry. [H]
Mette et al. (2016) [47]	USA	Patients receiving telegenetic (*n* = 56) or traditional (*n* = 63) GC for hereditary cancer risk	Satisfaction was high, with no difference between telegenetic or traditional GC. [F] Overall, 80% had GT and 49% had a decreased concern of developing cancer. [C, H]
Solomons et al. (2018) [48]	USA	New patients seen by genetics for personal/family history of cancer receiving telegenetic (*n* = 90) or traditional (*n* = 68) GC	Satisfaction in the telegenetic group was high. [F] Advantages included reduced travel and minimized time off work/child care needs. [F] Telegenetic GC was acceptable, but 13% reported that it did not address their needs. [F] Both groups had significant increases in HBOC knowledge immediately, and one month after GC, with no difference between groups. [G] Depression significantly reduced immediately after telegenetic GC, and anxiety significantly reduced immediately after traditional GC; both groups had reduced anxiety and depression one month after GC. [H] 32% of those who had telegenetic GC would have preferred traditional GC. [J]

GC = genetic counseling; GT = genetic testing; OC = ovarian cancer; BC = breast cancer; CRC = colorectal cancer; HBOC = hereditary breast/ovarian cancer. *publications from the same study.

**Table 3:** Group genetic counseling models.

Study	Country	Population	Selected Outcomes: GC Referral Rates [A], GC Attendance [B], GT Uptake [C], Wait Time [D], GC Time [E], Satisfaction [F], Knowledge [G], Psychosocial [H], Health Behaviors [I], Patient Preferences [J]
Mangerich et al. (2008) [49]	USA	Individuals interested in *BRCA* GT (*n* = 15; 6 with BC) attending a group education class	Time per patient decreased as the number of patients increased (estimated 125 min for individualized and 73 min for groups of six). [E] High satisfaction levels with the class. [F] Knowledge scores improved after the class. [G] The cost per patient decreased as the number of patients increased ($80 for individualized and $47 for groups of six).
Ridge et al. (2009) [50]	CAN	Women offered appointments for GC, including: group GC (*n* = 42), traditional GC (*n* = 37), OC patients receiving group GC (*n* = 10)	36% of women who were offered the general group GC session attended. [B] Length of GC was 75 min for the general group and 60 min for the OC group. [E] Satisfaction was high for group and traditional GC. [F] Some women found GC upsetting (20% group; 10% ovarian; 5% of control) or stressful (13% group; 10% ovarian; 11% control). [H] 40% of women who were offered group GC actively declined and attended traditional GC. [J]
Rothwell et al. (2012) [51]	USA	Women with or at high risk of BC/OC receiving group (*n* = 17) or traditional (*n* = 32) GC	Lower GT uptake in group (47%) compared to traditional (78%) GC. [C] GC time was shorter for group compared to traditional GC (35 vs. 63 min). [E] High satisfaction scores in both group and traditional GC. [F] Baseline knowledge was high, with no significant improvement in either model. [G] Significant increase in perceived personal control in group and traditional GC. [H] Decrease in cancer-specific distress in group and traditional GC, with significant reductions in intrusion (both) and in avoidance (traditional only). [H] Both groups had significant improvements in overall distress/depression, but not anxiety. [H] Most patients (65%) elected to receive traditional GC. [J]
Manchanda et al. (2016) [52]	UK	Randomized trial of Ashkenazi Jewish men/women without previous *BRCA* testing receiving group DVD (*n* = 409) or traditional (*n* = 527) GC	Uptake of GT was equivalent, with high uptake in group DVD (87%) and traditional (89%) GC models. [C] GC time was 20.5 min shorter for DVD GC. [E] DVD was non-inferior to traditional GC on patient satisfaction, patient knowledge, and risk perception. [F, G, H] DVD GC resulted in a decreased cost of £14/individual counseled.
Benusiglio et al. (2017) [53]	FRA	BC and OC patients eligible for *BRCA* GT receiving group (*n* = 210) or traditional (*n* = 47) GC	Individual GC time was shorter following group (18 min) compared to traditional (40 min) GC. [E] Satisfaction scores were high, with slightly higher scores for traditional GC. [F] Significant increase in knowledge after group GC; post-GC knowledge/knowledge improvement was inferior for traditional GC. [G]
Wiesman et al. (2017) [54]	USA	Ashkenazi Jewish men/women at low risk of a *BRCA* mutation receiving group GC (*n* = 88)	All eligible patients who attended GC consented to GT. [C] Most reported being comfortable learning in a group (97%), that the counselor explained the concepts (100%), and that they felt they could ask questions (100%). [F] 8% of patients would have preferred individualized GC. [F] Two *BRCA* mutation carriers were identified; both had risk-reducing surgery. [I]

GC = genetic counseling; GT = genetic testing; OC = ovarian cancer; BC = breast cancer.

**Table 4:** Genetics embedded models.

Study	Country	Population	Selected Outcomes: GC Referral Rates [A], GC Attendance [B], GT Uptake [C], Wait Time [D], GC Time [E], Satisfaction [F], Knowledge [G], Psychosocial [H], Health Behaviors [I], Patient Preferences [J]
Kentwell et al. (2017) [55]	AUS	Non-mucinous OC patients diagnosed <70 years before (*n* = 134) and after (*n* = 99) the implementation of embedded GC	GC referral rate increased from 54% to 85%. [A] 97% of newly diagnosed patients were referred for GC. [A] With the embedded GC model, the wait time for GC was 48 days, and the time from referral to GT result was 123 days. [D] Average face-to-face GC time decreased from 120 min to 52 min. [E]
Senter et al. (2017) [56]	USA	Newly diagnosed OC patients before (*n* = 401) and after (*n* = 336) the implementation of embedded GC	Increased referral rate from 21% to 44% in the first year; 50% in the second year. [A] Increased GC scheduling rate from 38% to 84%. [B] Highest rates of referral (51%) and patient follow-through (89%) when genetic counselors and oncologists were in the same oncology clinic on the same day. [A, B] No difference in uptake of GT before (96%) or after (97%) embedded process. [C] Wait times for GC decreased (2.52 months vs. 1.67 months); 25% of patients were seen on the date of referral with the embedded model. [D]
Bednar et al. (2017) [57] *	USA	OC patients (*n* = 1636) seen after implementation of embedded GC, mainstreaming GT, and GC-assisted referral processes	87% of patients referred for GC. [A] 8.6% declined GC. [C] Wait time for GC decreased from 197 days to 78 days *. [D]
Pederson et al. (2018) [58]	USA	Newly diagnosed BC patients before (*n* = 471) and after (*n* = 440) implementation of embedded GC	Patients were 49% more likely to be referred for GC. [A] Patients were 66% more likely to attend GC. [B] Wait time to GC decreased by 74%. [D] 69% were more likely to receive GT result prior to surgery. [D] 31% decrease in the time to treat BC. [D] No significant difference in surgical choices (mastectomy vs. lumpectomy). [I]

GC = genetic counseling; GT = genetic testing; OC = ovarian cancer; * outcomes are the result of multiple methods including embedded GC.

**Table 5:** Mainstreaming genetic testing models.

Study	Country	Population	Selected Outcomes: GC Referral Rates [A], GC Attendance [B], GT Uptake [C], Wait Time [D], GC Time [E], Satisfaction [F], Knowledge [G], Psychosocial [H], Health Behaviors [I], Patient Preferences [J]
George et al. (2016) [59]	UK	OC patients (*n* = 207) receiving *BRCA* GT via their oncology team	100% of *BRCA*+ were referred for and attended post-test GC. [B] 97% of OC patients consented to GT. [C] 4× reduction in patient wait times for GT. [E] Satisfaction was high; 98% of OC patients were happy that GT was done during an oncology appointment. [F] 0% wanted a separate appointment with a genetic counselor for pre-test GC. [J] 13× reduction in healthcare cost.
Bednar et al. (2017) [57]	USA	OC patients (*n* = 197) seen at regional oncology clinic	56% of OC patients who were seen at a regional clinic location had GT via their oncology team. [C]
Yoon et al. (2017) [60]	MAL	*Conference abstract:* OC patients (*n* = 208) receiving *BRCA* GT via their oncology team	Most patients were satisfied, not conflicted with their decision, and felt informed about their choices. [F] 79% of patients at pre-GT, and 69% at post-GT, had distress related to living with cancer; 41% had frequent concerns about getting cancer again at both time points. [H] Using the distress thermometer, some patients required psychosocial support at pre-GT (26%) and post-test GT (17%). [H]
Colombo et al. (2018) [61]	USAITAESP	OC patients (*n* = 634) receiving *BRCA* GT via their oncology team	The average time from blood sample to result was 4.7 weeks. [D] The average pre-test GC time with the oncology team was 20 min. [E] >99% of patients were satisfied with pre-test GC and post-test GC. [F] 94% were glad to have had testing at the time of their oncology appointment. [F]
Rahman et al. (2018) [62]	UK	OC patients (*n* = 122) receiving *BRCA* GT via oncology team	89% of *BRCA*+ were referred for GC, most 12–43 days after result disclosure. [A, D] 22% of *BRCA* VUS were referred for GC. [A] 100% GT uptake. [C] The time to GT result was 26 working days. [E]

GC = genetic counseling; GT = genetic testing; OC = ovarian cancer; VUS = variant of uncertain significance.

**Table 6:** Direct genetic testing models.

Study	Country	Population	Selected Outcomes: GC Referral Rates [A], GC Attendance [B], GT Uptake [C], Wait Time [D], GC Time [E], Satisfaction [F], Knowledge [G], Psychosocial [H], Health Behaviors [I], Patient Preferences [J]
Brierley et al. (2010) [63]	USA	Series of cases without pre-test GC (*n* = 21)	Three major patterns (wrong GT ordered, GT results misinterpreted, and inadequate GC) led to negative patient outcomes (unnecessary surgery, unnecessary GT, psychosocial distress, false reassurance, inappropriate medical management).
Metcalfe et al. (2010) [64] *	CAN	Ashkenazi Jewish women aged 25–80 years (*n* = 1516) pursuing *BRCA* GT using the direct GT model with written information	Overall satisfaction with GT was high, with no difference between *BRCA*+ and *BRCA*-women; 98% would recommend GT to other Ashkenazi Jewish women, including 94% of *BRCA*+. [F] *BRCA*+ women significantly increased their perceived BC risk (41% to 60%) and slightly decreased their perceived OC risks (36% to 26%). Perceived risks did not significantly change among *BRCA*− women. [H] Distress did not change among *BRCA*− women, but significantly increased among *BRCA*+ women; 56% of *BRCA*+ women had moderate–severe distress scores. [H] 19% overall (56% of *BRCA*+ and 18% of *BRCA*−) would have preferred face-to-face pre-test GC, and 22% of *BRCA*− would have preferred in-person results. [J]
Metcalfe et al. (2012) [65] *	CAN	Ashkenazi Jewish women aged 25–80 years identified to have a *BRCA* mutation via direct GT with written information (*n* = 19)	Significant declines in cancer-related distress were seen over time; average distress scores were 5.2 at baseline, 24 one year after, and 17 two years after GT. [H] One year after GT results, 100% had BC screening, and 95% had OC screening. [I] Two years after GT results, 11% had a risk-reducing mastectomy, and 94% of women >35 at the time of GT had a risk-reducing oophorectomy. [I]
Pal et al. (2014) [66]	USA	Women in the Inherited Cancer Registry database with *BRCA* mutation (*n* = 438)	GC was longer when a genetics professional was involved, 77% (vs. 21%) of pre-test GC sessions were >30 min. [C] Uptake of breast MRI was higher when *BRCA* testing/pre-test GC was completed by a genetic professional; uptake of contralateral risk-reducing mastectomy was higher in GC sessions >30 min and with a genetic professional. [I]
Armstrong et al. (2015) [67]	USA	Women who had *BRCA* GT with (*n* = 1334) or without (*n* = 2247) pre-test GC	Women who received pre-test GC reported greater knowledge (OR = 1.19) and satisfaction (OR = 2.56). [F, G]
Sie et al. (2014) [68] ^#^	NED	Women with BC referred for *BRCA* GT electing to have direct GT (*n* = 95) or pre-test GC (*n* = 66)	100% of direct and 76% of pre-test GC had GT. [C] Process time (triage to results) was lower in the direct group (70 days vs. 103 days); there was no difference in the time from GT to results (34 days). [D] Satisfaction with direct was high; most would choose this option again (89%) and recommend it to others (70%). [F] Women opting for pre-test GC had higher decisional conflict and baseline distress; there were no differences in quality of life, BC worry, and risk perception of second BC or hereditary cancer among women who chose direct GT or pre-test GC. [H]
Sie et al. (2016) [69] ^#^	NED	Women with BC referred for *BRCA* GT electing to have direct GT (*n* = 59, incl 5 *BRCA*+) or pre-test GC (*n* = 49, incl 1 *BRCA*+)	All participants were satisfied with their choice of GT method; 85% of direct would choose direct again, and 80% of pre-test would choose pre-test again. [F] Those in the direct group had lower distress scores, with greater differences in general distress at baseline than follow-up. [H] BC-specific distress (not hereditary-specific distress) was lower in direct GT. [H] Quality of life, BC worry, and risk perception for hereditary BC or second BC did not differ between groups or over time. [H] Direct GT were more likely to perceive heredity as a cause of BC (46% vs. 10%). [H]
Plaskocinska et al. (2016) [70] ^##^	UK	Women with a recent (<12 months) diagnosis of OC (*n* = 173) who had direct *BRCA* GT	Acceptability of GT was high, and women felt they had enough information and time to decide to pursue GT. [F] Distress and depression/anxiety/stress scores in response to GT were significantly lower than the equivalent scores in response to OC diagnosis. Younger women had more intrusive thoughts and stress; *BRCA*+ women had higher levels of cognitive avoidance. [H] The cost per patient was less than the traditional GC (£243 vs. £383).
Shipman et al. (2017) [71] ^##^	UK	*Qualitative Study* of women diagnosed with OC in last 12 months with positive (*n* = 4), negative (*n* = 5) and inconclusive (*n* = 3) GT results from direct *BRCA* GT	Motivations/influences included: GT was not disruptive in the context of OC diagnosis, social/family considerations were persuasive. [H] Impacts included reassurance for most participants who received negative results, trading uncertainties for those who received inconclusive results, managing finding an uncertain or pathogenic variant, and the added responsibility of notifying family members. [H]
Meiser et al. (2016) [72] ^	AUS	*Qualitative Study of* women aged 18–49 years with BC who received *BRCA* GT with pre-test GC or direct GT with written information who were *BRCA*+/fhx+ (*n* = 5) *BRCA*+/fhx− (*n* = 5), *BRCA*−/fhx+ (*n* = 5), or *BRCA*−/fhx− (*n* = 5)	Most were grateful for GT, and did not find that it added stress to their diagnosis. [H] BRCA+/fhx+ expected the result, while *BRCA*+/fhx− were surprised. BRCA−/fhx+ felt a lack of closure, and *BRCA*−/fhx− were relieved. [H] All *BRCA*+ and 1/5 *BRCA*−/fhx+ had bilateral mastectomies. [I] Advantages of *BRCA*+ were easing the burden of treatment and awareness of familial risk. Advantages of *BRCA*− was alleviation of worry. Most reported no disadvantages to GT, but suggested more a formal follow-up after acute treatment was complete.
Quinn et al. (2017) [73] ^	AUS	Women aged 18–49 years with BC who received treatment focused *BRCA* GT with pre-test GC (*n* = 70) or direct GT with written information (*n* = 65)	Written information was non-inferior to pre-test GC on knowledge, cancer-specific distress, anxiety and depression, test-related distress, positive test experience, or family involvement. [G, H] Decisional conflict with written information was non-inferior to pre-test GC. [H] There was no difference in decision regret about GT or risk-reducing surgery. [H] There was no difference in the update of bilateral mastectomy or risk-reducing oophorectomy at one year. [I] The cost/women counseled in the written group ($89AUD) was lower than pre-test GC ($173AUD).
Høberg-Vetti et al. (2016) [74] ^^	NOR	Women offered direct *BRCA* GT with written information after a new diagnosis of BC (*n* = 893) or OC (*n* = 122)	45% of breast and 68% of ovarian cancer patients pursued GT. [C] The proportion of patients with high (≥8) anxiety scores decreased between baseline (40%), one week (24%), and six months (20%) after GT results. [H] Approximately 10% had high depression scores at all of the time points. [H] No significant differences in depression and anxiety scores between the following groups: breast vs. ovarian cancer; mutation carriers vs. non-mutation carriers; GT > 90 days vs. <90 days after diagnosis. [H]
Augestad et al. (2017) [75] ^^	NOR	*Qualitative study* of women newly diagnosed with BC (*n* = 13) or OC (*n* = 4) who received direct *BRCA* GT with written information	Themes included: being beside oneself after a cancer diagnosis, altruism, and ethical dilemmas related to GT, and the need for support and counseling to assist the decision process. [H] Acceptance of GT was primarily motivated to protect their children. [H] Women preferred a conversation with a health provider prior to GT. [J]
Lieberman et al. (2017a) [76] **	ISR	Ashkenazi Jewish individuals aged ≥25 years who were self- (*n* = 744) or recruiter (*n* = 1027) enrolled for direct *BRCA* GT with written information	100% of BRCA+ and 87% of *BRCA*− with family history attended post-test GC. [B] Satisfaction was high, with higher satisfaction among self-referral and those with higher perceived personal control. There was no difference in satisfaction between *BRCA*+ and *BRCA*−; overall, 90% would recommend BRCA screening. [F] Satisfaction with health decisions was high and increased after GT results were disclosed, with higher scores in the self-referral group, and no difference based on *BRCA* status. [F] Knowledge levels were high, with higher levels among self-referred, *BRCA*+, and *BRCA*− with family history. [G] Perceived personal control was higher after GT, irrespective of BRCA status. [H] Cancer-related distress was low, with lower distress in the self-referred group and men; *BRCA*+ had significantly higher levels of distress than *BRCA*− (19.9 vs. 4.9). [H] State anxiety was slightly higher among the self-referred, with no significant change over time; *BRCA*+ had higher levels of anxiety (12.6 vs. 9.9). [H]
Lieberman et al. (2017b) [77] **	ISR	*Qualitative Study* of Ashkenazi Jewish individuals ≥25 who were *BRCA*+ (*n* = 26) or *BRCA*− (*n* = 10) after direct GT with written information	81% of *BRCA*+ and 90% of *BRCA*− had a positive attitude toward GT. [F, H] Motivations for GT were to know risk status and have an accurate risk assessment. Barriers were lack of physician awareness/support, lack of public awareness, coping with knowing. [H] 79% of *BRCA*+ preferred streamlined screening to traditional GC. [J] All would recommend universal screening for Ashkenazi Jewish population; a few thought that pre-test GC should be provided. [J]

GC = genetic counseling; GT = genetic testing; BC = breast cancer; OC = ovarian cancer; fhx = family history; * publications from the same study, ** publications from the same study; ^ publications from the same study, ^^ publications from the same study; ^#^ publications from the same study, ^##^ publications from the same study.

**Table 7:** Tumor-first genetic testing models.

Study	Country	Population	Selected Outcomes: GC Referral Rates [A], GC Attendance [B], GT Uptake [C], Wait Time [D], GC Time [E], Satisfaction [F], Knowledge [G], Psychosocial [H], Health Behaviors [I], Patient Preferences [J]
**Studies of tumor screening for Lynch Syndrome:**			
Landsbergen et al. (2012) [78]	NED	Recently diagnosed CRC < 50 OR second CRC < 70 years old (*n* = 400) who had tumor screening.	There were no significant differences in cancer-related distress between those with normal or abnormal tumor results. Immediately after results, 40% of those with abnormal results had high cancer-related distress. [H] Perceived risk of CRC increased over time for both groups, with an overall increase from 43% to 50%. [H] There were no differences in mood states or social support between the groups. [H]
Heald et al. (2013) [79]	USA	CRC where universal screening results went only to the surgeon (*n* = 237), to the surgeon and genetics (*n* = 87), and to the surgeon and genetics with a genetic counselor contacting the patient (*n* = 784)	GC referral rates improved with the increased involvement of genetic counselors (55% with no involvement; 82% with receipt of results only; 100% with direct patient contact). [A] Of those referred, uptake of GC increased with the increased involvement of genetic counselors (57% vs. 78% vs. 71%). [B] Overall, 88% of those receiving GC consented to GT. [C]
Marquez et al. (2013) [80]	USA	Universal tumor screening of CRC ≤ 70 years old (*n* = 129)	100% of patients with abnormal tumor results were referred to GC. [A] 92% (11/12) of those referred had GC and appropriate follow-up; 1/12 did not have insurance coverage. [B, I] Of those seen for GC, uptake of GT was 100%. [C]
Moline et al. (2013) [81]	USA	Universal tumor screening of EC patients (*n* = 245)	68% of abnormal were referred for GC. [A] 76% had GC and 83% pursued GT. [B, C]
Ward et al. (2013) [82]	AUS	CRC patients with mismatch repair deficient tumors (*n* = 245)	18% of patients were referred for GC: low risk (2.5%) < high risk (43%) Factors predicting referral were high risk of Lynch syndrome, young age at diagnosis, and right-sided tumor. [A] 89% attended GC: low risk (67%) < high risk (90%). [B] 87% had GT: low risk (50%) < high risk (90%). [C] 69% of those who did not have clinical GT provided research samples, two additional mutations were found: one disclosed, and one declined to receive research results. [C]
Batte et al. (2014) [83]	USA	Universal screening of unselected EC (retrospective = 408; prospective = 206)	The prospective group had higher rates of GC (72%) than the retrospective group (42%); GT rates were similar (77% prospective and 79% retrospective). [B, C] Facilitators to GC were a younger age and had a recent diagnosis; barriers included a perceived lack of relevance, inability to travel, limited insurance, and previous GC/GT. [B]
Hall et al. (2014) [84]	USA	Consecutive CRC and EC patients who had reliable internet access (*n* = 66) whose tumor screening results were disclosed directly via their electronic medical record	Feasibility was reached as 74% of patients consented to receive tumor results on the electronic medical record, and 86% viewed the result Most patients with normal (56%) and abnormal (60%) tumor results reported speaking to their doctor. Of five patients with abnormal results, three contacted clinical genetics, and one pursued GT via medical oncology. [B, C, I] Acceptability and satisfaction were high regardless of tumor results, with higher rates among those with a higher perceived risk of hereditary cancer. [F] Perceived hereditary risks were described as low–moderate. [H] No differences in anxiety at baseline vs. 72 h after the GT results, but anxiety was significantly lower one month after results. [H] No difference in anxiety between abnormal and normal tumor results. [H]
Frolova et al. (2015) [85]	USA	EC before (*n* = 395) and after (*n* = 242) the implementation of universal tumor screening	100% of patients with abnormal tumor results were referred for GC pre-implementation and post-implementation of universal tumor screening. [A] Higher rates of patient acceptance for GC post-universal screening (95%) compared to pre-universal screening (63%); rates of GT were similar (71% pre and 76% post). [B, C] Among patients with normal tumor screening, 12% of were referred for GC in both the pre-universal testing and post-universal testing groups, with no significant difference in the proportion of women who were offered and underwent GT. [A, C] Overall, significantly more women underwent GT after the implementation of universal screening (9.1% vs. 4.8%). [C]
Kidambi et al. (2015) [86]	USA	CRC in selected (<50 or <60 with features of Lynch syndrome; *n* = 107) and universal screening groups (*n* = 285)	100% of selected and 92% of universally screened patients with abnormal tumor results were contacted by a genetic counselor. [A] Uptake of GC was lower in the universal (64%) compared to selective (87%) group; however, the uptake of GT was higher in the universal group (93% vs. 77%). [B, C]
Hunter et al. (2015) [87]	USA	CRC patients undergoing universal tumor screening (*n* = 145)	35% worried about having a gene mutation; these patients were younger or had lower education levels. [H] >90% of patients agreed they would be able to cope with their results, that the test should be available, and they understood the reason for the test. [H] Overall distress scores were low; 77% had a lack of distress, and 2% had high distress, which was not associated with age, stage, perceived risk, or endorsement of benefits/barriers. [H] 93% of participants intended to share their results with their health care providers, with women being more likely to share than men. [I]
Goverde et al. (2016) [88]	NED	Consecutive series of EC patients ≤70 years (*n* = 179) having tumor screening	100% of patients with abnormal results were offered GC. [A] 100% of patients received GC, and 91% consented to GT. [B, C] Routine Lynch syndrome screening in EC patients ≤ 70 years is cost-effective.
Brennan et al. (2017) [89]	AUS	Consecutive series of CRC patients (*n* = 1612) having tumor screening	30% of patients with abnormal tumor results were seen for GC. Facilitators were younger age (<50), mucinous histology, and practitioner (higher referral rates in surgeons and oncologists). [B]
Holter et al. (2017) [90]	CAN	*Conference abstract:* CRC cancer patients <60 years (*n* = 502) undergoing tumor screening	100% referred for GC; 94% received GC, and of those, 90% had GT. [A, B, C] Time from referral to GC was 59 days, and referral to GT result was 103 days. [D]
Hunter et al. (2017) [91]	USA	Newly diagnosed CRC patients (*n* = 189) undergoing tumor screening	92% of patients with abnormal tumor results were interested in GT and 87% pursued additional GT to confirm their risks. [C] Attitudes of tumor testing were positive, especially in those who worried that they had Lynch syndrome and who endorsed more benefits to tumor testing. Most patients (93%) wanted to know if they were at risk of hereditary CRC. [H] Prior to results, patients intended to share results with children/sibling (96%), parents (89%), or any relative (84%). Of patients with abnormal results, 93% reported sharing results with at least one first-degree relative. The most common reason to share was a sense of responsibility, and the more common barrier was worrying that their relatives would worry about getting cancer. [H, I]
Kupfer et al. (2017) [92]	USA	*Conference abstract:* CRC in White (*n* = 266) African American (*n* = 174), and Hispanic (*n* = 125) patients having tumor screening	Caucasian patients were more likely to be referred for GC (64%) compared to African American (54%) and 21% of Hispanic patients. [A] Of those who had GC, 80% of Caucasian, 71% of African American, and 40% of Hispanic patients had GT. [C]
Livi et al. (2017) [93]	ITA	*Conference abstract:* Consecutive EC patients (*n* = 166) having tumor screening	97% of those with abnormal results had GT; 2% declined. [C]
Najdawi et al. (2017) [94]	AUS	Patients with EC (any histology) and endometroid or clear cell gynecological cancer (*n* = 124) having tumor screening	100% of those considered at high risk of lynch syndrome were referred for GC, and 82% had GT. [A, C]
O’Kane et al. (2017) [95]	IRL	CRC patients having tumor screening at one of three centers (*n* = 3906)	Overall, 56% of patients with abnormal tumor testing were not referred to genetics, declined, or did have additional tumor testing; the highest rate of referral (66%) was noted a center using a physician-requested model compared to two centers using universal screening models (33% and 30%). [A] Of those referred for GC, 11% declined or did not attend. [B]
Patel et al. (2017) [96]	USA	*Conference abstract:* Consecutive CRC patients (*n* = 1597) having tumor screening	100% of patients who screened positive were referred for GC. [A] Of those referred for GC, 77% had GC, and 88% had GT. Non-completion of GC and GT was associated with older age and a lack of private insurance. [B, C] Of those tested, 83% were enrolled in a high-risk clinic. [I]
Watkins et al. (2017) [97]	USA	EC patients (*n* = 242) having tumor screening	91% of those who had GC, pursued GT. [C]
Martin et al. (2018) [98]	USA	Newly diagnosed CRC patients (*n* = 78) having tumor screening	Referral for GC was submitted 70.5% of the time, with CRC surgeons more likely to refer than general surgeons. [A] 27% of referred patients did not complete GT. [C] Wait time to GC referral was 65 days; GC referral to first visit was 71 days. [D]
Metcalfe et al. (2018) [99]	USA	Consecutive upper tract urothelial cancer patients (*n* = 115) having tumor screening	100% of patients who screened positive were referred for GT. [A] 56% of patients/families received GC and GT. [B, C]
Miesfeldt et al. (2018) [100]	USA	CRC (*n* = 175) or EC (*n* = 276) patients where results were sent to a surgeon, patient navigator, or both	30 patients (16 navigated, 14 non-navigated) had abnormal tumor results. 100% of navigated patients were referred; 88% received GC, and 100% of those eligible had GT. 42% of non-navigated patients received GC, and 80% of those eligible had GT. [A, B, C]
**Studies of tumor genetic testing**			
Gray et al. (2016) [101]	USA	Patients with stage IV lung or CRC (*n* = 167) enrolled in a tumor testing study	Genetic knowledge was moderately low. [G] Patients had positive attitudes about having a genetic test. [H] >95% patients chose to learn about cancer-related, pharmacogenetic, and carrier status results. Most also wanted to know about negative prognostic results (84%) and the risk of developing an untreatable non-cancer condition (85%). [J] Patients with less positive attitudes about GT were less likely to have a high preference for the return of GT results. [J]
Pinheiro et al. (2017) [102]	USA	Cancer patients being offered or receiving tumor results (*n* = 66)	Patients’ top two preferred information topics were the benefits of tumor GT (88%) and how tumor GT determines treatment (88%); 71% were interested to hear about implications for family members. [J] Patients preferred to receive information from their nurse/physician (85%) or written information (67%), few preferred the internet (29%) or short videos (12%). [J]
Best et al. (2018) [103]	AUS	Patients with advanced solid tumors participating in a molecular tumor screening study (n > 369)	Protocol of a mixed-methods longitudinal study examining psychosocial and ethical issues and outcomes in germline genomic sequencing for cancer. Planned evaluation of patient knowledge, satisfaction, preferences for genetic information, attitudes about tumor genomic profiling, and the behavioral, decisional, and psychological outcomes and their respective predictors. [F, G, H, I, J]

GC = genetic counseling; GT = genetic testing; CRC = colorectal cancer; EC = endometrial cancer.

## Appendix A. Medline Search Strategy

1	exp Neoplasms/
2	neoplas*.mp,kw.
3	paraneoplas*.mp,kw
4	cancer*.mp,kw.
5	tumo?r*.mp,kw.
6	onco*.mp,kw.
7	metast*.mp,kw.
8	malignan*.mp,kw.
9	adenocarcin*.mp,kw.
10	carcin*.mp,kw.
11	HBOC.mp,kw.
12	HNPCC.mp,kw.
13	lynch*.mp,kw.
14	or/1-13
15	exp Genetic Services/
16	Genetic Counseling/
17	Genetic Testing/
18	Genes, Neoplasm/
19	Germ-Line Mutation/
20	(genetic? adj2 service?).mp,kw.
21	(genetic? adj2 counsel*).mp,kw.
22	(genetic? adj2 screen*).mp,kw.
23	(genetic? adj2 test*).mp,kw.
24	(somatic adj2 screen*).mp,kw.
25	(somatic adj2 test*).mp,kw.
26	(germline? adj2 screen*).mp,kw.
27	(germline? adj2 test*).mp,kw.
28	(profiling* adj2 screen*).mp,kw.
29	(molecular* adj2 screen*).mp,kw.
30	or/15-29
31	(universal* adj3 screen*).mp,kw.
32	14 and 31
33	“Outcome and Process Assessment (Health Care)”/
34	“Outcome Assessment (Health Care)”/
35	exp Patient Outcome Assessment/
36	Patient Reported Outcome Measures/
37	“Early Detection of Cancer”/
38	Incidental Findings/
39	Psychological Trauma/
40	exp Stress Psychological/
41	Stress Disorders, Traumatic/
42	Stress Disorders, Post-Traumatic/
43	Stress Disorders, Traumatic, Acute/
44	exp Patient Satisfaction/
45	Patient Preference/
46	Patient Access to Records/
47	(patient? adj3 outcome?).mp,kw.
48	(clinc* adj3 outcome?).mp,kw.
49	(counsel* adj3 outcome?).mp,kw.
50	(earl* adj3 detect*).mp,kw.
51	(profiling* adj2 detect*).mp,kw.
52	(incidental* adj2 finding?).mp,kw.
53	(incidental* adj2 germline?).mp,kw.
54	(psycho* adj3 trauma*).mp,kw.
55	(psycho* adj3 measur*).mp,kw.
56	(psycho* adj3 outcome?).mp,kw.
57	(psycho* adj3 function*).mp,kw.
58	(psycho* adj3 impact*).mp,kw.
59	(psycho* adj3 effect?).mp,kw.
60	(psycho* adj3 distress*).mp,kw.
61	(patient? adj2 satisfact*).mp,kw.
62	(patient? adj2 prefer*).mp,kw.
63	access*.mp,kw.
64	mainstream*.mp,kw.
65	main-stream*.mp,kw.
66	universal*.mp,kw.
67	model?.mp,kw.
68	or/33-67
69	14 and 30 and 68
70	32 or 69
71	exp animals/not (exp animals/and exp humans/)
72	70 not 71
73	limit 72 to “all child (0 to 18 years)”
74	limit 72 to “all adult (19 plus years)”
75	73 not 74
76	72 not 75
77	limit 76 to yr = “2007-Current”
78	limit 77 to English language
79	remove duplicates from 78

## Appendix B. Study Inclusion and Exclusion Criteria

**Inclusion**	**Exclusion**
1. English studies 2. Adults with cancer 3. Published ≥ 2007 4. Conference abstracts published ≥ 2015 5. Involve alternative models of genetic service delivery including: - alternative provider (i.e., physician ordered) - alternative test protocol (i.e., tumor testing or tumor screening) - alternative mode of pre-test genetic counselling	1. Direct-to-consumer genetic testing 2. Germline whole exome sequencing 3. Genetic testing for non-cancer indications 4. Tumor testing for somatic targets only 5. Single nucleotide polymorphism testing only 6. Studies not considering patient impact of alterative genetic testing, including: - Detection rates ONLY - Clinician impact/perception ONLY - Summary of available methods without assessment - Cost-effectiveness ONLY - Mutation rates ONLY - Studies of implementation planning ONLY
